# Working Memory Training in Amnestic and Non-amnestic Patients With Mild Cognitive Impairment: Preliminary Findings From Genotype Variants on Training Effects

**DOI:** 10.3389/fnagi.2021.624253

**Published:** 2021-02-15

**Authors:** Susanne S. Hernes, Marianne M. Flak, Gro C. C. Løhaugen, Jon Skranes, Haakon R. Hol, Bengt-Ove Madsen, Anne-Brita Knapskog, Andreas Engvig, Are Pripp, Ingun Ulstein, Trine Lona, Xin Zhang, Linda Chang

**Affiliations:** ^1^Department of Geriatric and Internal Medicine, Sørlandet Hospital, Arendal, Norway; ^2^Department of Clinical Science, University of Bergen, Bergen, Norway; ^3^Department of Clinical and Molecular Medicine, Norwegian University of Science and Technology, Trondheim, Norway; ^4^Department of Pediatrics, Sørlandet Hospital HF, Arendal, Norway; ^5^Department of Radiology, Sørlandet Hospital HF, Arendal, Norway; ^6^Department of Geriatric Medicine, The Memory Clinic, Oslo University Hospital, Oslo, Norway; ^7^Department of Medicine, Diakonhjemmet Hospital, Oslo, Norway; ^8^Oslo Centre of Biostatistics and Epidemiology Research Support Services, Oslo University Hospital, Oslo, Norway; ^9^Department of Psychiatry, Age Psychiatry, The Hospital of Telemark, Skien, Norway; ^10^Department of Diagnostic Radiology and Nuclear Medicine, and Department of Neurology, University of Maryland School of Medicine, Baltimore, MD, United States; ^11^Department of Neurology, Johns Hopkins University School of Medicine, Baltimore, MD, United States; ^12^Department of Medicine, John A. Burns School of Medicine, The University of Hawai‘i at Mānoa, Honolulu, HI, United States

**Keywords:** MCI, Alzheimer, working memory training, *APOE* genotype, LMX1A

## Abstract

Working memory training (WMT) effects may be modulated by mild cognitive impairment (MCI) subtypes, and variations in *APOE*-epsilon (*APOE*-ε) and *LMX1A* genotypes. Sixty-one individuals (41 men/20 women, mean age 66 years) diagnosed with MCI (31 amnestic/30 non-amnestic) and genotyped for *APOE*-ε and *LMX1A* completed 4 weeks/20–25 sessions of WMT. Cognitive functions were assessed before, 4 weeks and 16 weeks after WMT. Except for Processing Speed, the non-amnestic MCI group (naMCI) outperformed the amnestic MCI (aMCI) group in all cognitive domains across all time-points. At 4 weeks, working memory function improved in both groups (*p* < 0.0001), but at 16 weeks the effects only remained in the naMCI group. Better performance was found after training for the naMCI patients with *LMX1A*-AA genotype and for the *APOE*-ε4 carriers. Only the naMCI-*APOE*-ε4 group showed improved Executive Function at 16 weeks. WMT improved working memory and some non-trained cognitive functions in individuals with MCI. The naMCI group had greater training gain than aMCI group, especially in those with *LMX1A*-AA genotype and among *APOE-*ε4-carriers. Further research with larger sample sizes for the subgroups and longer follow-up evaluations is warranted.

## Introduction

Yearly, as many as 15% of individuals with mild cognitive impairment (MCI) transition into dementia (Huang et al., 2016). Delaying the onset of dementia by a mere 1 year alone can lead to one million fewer cases of incident dementia by 2050 (Zissimopoulos et al., 2014). Working memory (WM) deficits are often found in aging individuals, especially in those with MCI or early stages of Alzheimer’s disease (Huntley and Howard, 2010; Saunders and Summers, 2010). Cognitive training programs within the restorative paradigm is designed for targeting core cognitive functions, including WM. While WM capacity is limited, it can be expanded by training, with corresponding changes in neural mechanisms that underly this effect (Constantinidis and Klingberg, 2016). Randomized controlled trials indicate that computerized working memory training (WMT) may improve performance on WM tasks with similar processing demands (Simons et al., 2016). However, studies on computerized cognitive training programs in MCI individuals showed mixed results, only some studies showed benefits on cognition, and the transfer effects to non-trained tasks were inconclusive (Belleville et al., 2006; Rozzini et al., 2007; Talassi et al., 2007). The heterogeneity within the MCI population, due to various underlying brain pathology or co-morbid conditions, also might have contributed to the diverse findings on training effects of cognitive interventions in MCI patients.

Dopamine is involved in various brain functions, including arousal, motivation and higher executive functions. Dopaminergic function is also essential to cognition by regulating attention and mediating WM function (Goldman-Rakic, 1996a; Salami et al., 2019), as well as motor function and processing speed (Eckart and Bunzeck, 2013). However, both dopamine transporters and receptors, hence the dopaminergic synapses, decline with normal aging (van Dyck et al., 1995; Karrer et al., 2017), and in those with MCI, leading to psychomotor slowing, working memory deficits and parkinsonism in some individuals (Sasaki, 2018). Dopaminergic synapses hold a key role in plasticity (Söderqvist et al., 2012), and the loss of dopaminergic receptors is believed to be responsible for many adverse effects of cognitive aging (Li et al., 2010). The Lim homeobox transcription factor 1 alpha (*LMX1A*) gene is involved in the production, differentiation and preservation of dopaminergic neurons in the midbrain and is necessary for the brain’s development and maintenance of dopaminergic neurons. The association of allelic frequencies of the *LMX1A* and neurological diseases has been studied only to a limited extent (Bergman et al., 2010; Rolstad et al., 2015). For instance, *LMX1A*-AA carriers with HIV-associated neurocognitive disorders showed greater WM training gain than non-carriers (Chang et al., 2017), whereas conflicting results exist in cognitively normal individuals (Bellander et al., 2011, 2015).

Besides age, apolipoprotein E (*APOE*) is one of the most studied factors associated with cognitive decline. *APOE* carries phospholipids and cholesterol within the body and plays a major role in neuronal cholesterol metabolism and synaptogenesis. The *APOE-*ε4 allele has significant influence on cognitive function, and *APOE-*ε4 homozygosity is the strongest known single risk factor for late onset Alzheimer’s dementia. Individuals with *APOE-*ε4 have reduced synaptic plasticity (Arendt, 2009) and possibly impaired cognitive trainability. While individuals at younger or middle ages showed positive effects, those older than 65 years of age typically showed negative effects of the *APOE*-ε4 allele on cognitive performance (Chang et al., 2011); however, greater lifetime levels of cognitive activities seem to attenuate these negative effects (Wirth et al., 2014).

This sub-study of the Memory Aid study (Flak et al., 2014) investigated the effects of computerized WMT in patients with amnestic (aMCI) and non-amnestic MCI (naMCI). As a pilot study, we additionally explored the modulatory effects of allelic variations in *APOE*ε and *LMX1A* on the WM training outcomes. Since individuals with better WM capacity showed better cognitive training efficacy (Matysiak et al., 2019), we hypothesized that: (1) individuals with naMCI, who likely would have better WM capacity (Constantinidou et al., 2014), would display better cognitive performance and greater training effects, than those with aMCI; (2) based on the greater training effects in those with *LMX1A*-AA, both in healthy individuals (Bellander et al., 2011) and in those with a degenerative brain disorder (Chang et al., 2017), we also expected our MCI participants with *LMX1A*-AA genotype to show greater training effects than non-carriers; (3) based on the better cognitive performance in younger and middle age individuals with *APOE*-ε4 allele (Chang et al., 2011; Zink et al., 2019), we further expected our MCI participants (average age in the 60s) with at least one copy of this allele to show better training effects.

## Materials and Methods

The Norwegian Regional Committee for medical and health research ethics, South-Eastern region (2013/410) approved the study (clinicaltrials.gov NCT01991405). Written informed consent was obtained from each participant before study initiation. The data presented in this paper is a substudy of the Memory Aid study as described in the published protocol (Flak et al., 2014).

Participants were recruited from four Norwegian memory clinics and included in the study only if they fulfilled these inclusion criteria: (1) prior diagnosis of MCI within the last 15 months. The Petersen/Winblad criteria for MCI (Winblad et al., 2004) were used for diagnosis, as specified by the guidelines from the Norwegian registry of patients assessed for cognitive symptoms (NorCog). The assessment included neuropsychological tests, and questionnaires for ascertainment of risk factors. (2) Their willingness to complete the 20–25 session of WMT program and the follow-up evaluations. The participants were excluded from the study if they had any of these conditions: (1) any psychiatric conditions including depression; specifically, none of the participants had moderate or severe depression according to their pre-trial screenings; (2) history of significant brain disorders (e.g., stroke or epilepsy); (3) use of any type of dementia-delaying medication; (4) head trauma with post-traumatic loss of consciousness for at least 30 min during the lifespan; (5) loss of senses that might confound the training effects (e.g., blindness, deafness); (6) individuals with contraindications for magnetic resonance imaging (e.g., implanted metal foreign objects or severe claustrophobia), which was needed to exclude those with significant MRI lesions (e.g., prior strokes, tumors).

Socioeconomic status (SES) was assessed with Hollingshead’s index of education and occupational position, scaled from 1 (low) to 5 (high) (Hollingshead and Redlich, 2007).

### Working Memory Training (WMT)

The participants were randomized to 20–25 session/4 weeks of either adaptive or non-adaptive WMT using Cogmed^®^ RM (Pearson education, Inc.). The physical appearance of the cognitive training program is identical in the two versions of the program. In the adaptive version, the tasks became increasingly complex and difficult as the individual mastered each level, making the participant work at his or her maximum capacity at all times (i.e., adaptive training). In the non-adaptive version, the participants trained at a fixed low level of difficulty, with a span of three or fewer items per task. The training is described in detail elsewhere (Flak et al., 2019). However, since we did not observe group differences in the two types of training, and we have a limited sample size, we combined the participants who had the two training types into one group for each of the MCI subtype groups. Since the adaptive training would require the participants to master each level before they advanced to the next more difficult level, the lack of group differences in the adaptive versus non-adaptive training suggest that those who performed the adaptive training might have stayed at similarly low levels as those who performed the fixed lower level training.

### Neuropsychological Assessment

The participants were assessed with neuropsychological tests at three time points: at baseline (T0), 4 weeks after training (T1) and 16 weeks after training (T2). The cognitive evaluation included the administration of standardized and commonly used neuropsychological tests (Wechsler Memory Scale 3rd edition/WMSIII, Delis–Kaplan Executive Function System/D-KEFS, California Verbal Learning Test 2nd edition/CVLT-II, and Rey Complex Figure Test/RCFT). The tests were grouped into nine cognitive domains. Theoretical framework, clinical practice and research studies do not provide consensus on how neuropsychological tests should be clustered. Due to our small sample size, using factor analysis to cluster the tests into cognitive domains was not feasible. The tests were thus grouped into nine cognitive domains based on Rog and Finks recommendations for evaluating MCI (Table 1) (Rog and Fink, 2013). For Digit span, California Verbal Learning Test-II and Verbal Fluency alterative versions of the tests were used at each time-point to minimize practice effects. In order to compare cognitive performance across the domains, *Z*-scores were calculated based on the group performance at baseline as described elsewhere (Flak et al., 2019).

**TABLE 1:** Assessed cognitive domains and corresponding neuropsychological tests.

Cognitive domains	Tests
	
Working memory domain	WMS-IV digit span backward, WMS-III spatial span backward, WMS-III letter-number sequencing
Attention domain	WAIS-IV digit span forward, WMS-III spatial span forward, CVLT-II trial 1, CVLT-II trial B
Processing speed domain	WAIS-IV coding, WAIS-IV symbol search, D-KEFS color word interference test 1 color naming, D-KEFS color word interference test 2 word reading
Visual episodic learning/short delay recall domain	RCFT immediate recall, WMS-III faces I
Visual episodic memory/long delay recall domain	RCFT delayed recall, WMS-III faces II delayed recall
Verbal episodic learning/short delay recall	WMS-III logical memory I, CVLT-II total learning, CVLT-II short delay free recall
Verbal episodic memory/long delay recall verbal episodic memory, recognition	logical memory II delayed recall, CVLT-II long delay free recall, CVLT total hits CVLT total hits
Executive functions	RCFT copy trial, D-KEFS color word interference test 3 inhibition, D-KEFS color word interference test 4 inhibit/switching, D-KEFS verbal fluency test letter fluency, D-KEFS verbal fluency test category fluency, D-KEFS verbal fluency test category switching

*WAIS-IV, Wechsler Adult Intelligence Scale 4th ed.; WMS-III, Wechsler Memory Scale 3rd ed.; D-KEFS, Delis–Kaplan Executive Function System; RCFT, Reys Complex Figure Test; CVLT-II, California Verbal Learning Test 2nd ed.*

### Classification of Amnestic and Non-amnestic MCI Subtypes

Classification of the MCI subtype was performed after inclusion, according to the patient’s cognitive profiles at baseline. Individuals with scores more than −1.5 SD from the mean compared to norms on the delayed verbal and/or visual episodic memory were classified into the amnestic MCI (aMCI) group. Those with normal scores in the memory domains, combined with scores more than −1.5 standard deviation from the mean in one or more of the other domains assessed, were categorized into the non-amnestic MCI (naMCI) group (Petersen et al., 1999; Winblad et al., 2004).

### Genotyping/DNA Collection

DNA was extracted from saliva collected in Oragene Self collection Kit (DAN Genotek, Inc., Ottawa, ON, Canada). Genomic DNA was analyzed with Restriction Fragment Length Polymorphism (RFLP-PCR) for genotype analyses of *APOE*ε (rs429358 and rs7412) and *LMX1A* (rs4657412), as reported previously (Chang et al., 2016, 2017). Specifically, for *LMX1A*, genomic DNA were amplified by PCR using the primers LMX-5′:5′-CTCGCCTCCAGGAA TGGGTGTTGTA-3′ and LMX-3′: 5′-GCCACGAGGAACTTGTGAGAGGGTT-3′ for *LMX1A*, and APO-5′ and APO-3′ for *APOE*ε. The amplifications were performed on the denatured DNA (94°C for 5 min, 30 cycles at 94°C, annealed at 64°C for 30 s, and extending at 72°C for 30 s). The amplified PCR products were then digested with three restriction enzymes sequentially overnight at 37°C. The digested PCR products were then evaluated on 4% agarose gel and visualized using GelGreen^TM^ Nucleic Acid Gel Stain (89139–144, Biotium, Hayward, CA, United States).

### Statistical Analysis

Statistical analyses were performed with R version 3.5.2. Since no group differences on the training effects were found at baseline between the non-adaptive and adaptive WMT groups (Flak et al., 2019), the data from these two training groups were pooled and analyzed as one training group. The sample size and power calculations were based on the primary outcome of the Memory Aid study with differences in training gains between the adaptive and non-adaptive WMT as reported (Flak et al., 2019). Imputation was performed for two datapoints, for one subject at 4-week post training, and another at 16 weeks post training, using ‘missForest’ in R.

A weighted-general estimation model (WGEE) was used, with training (across baselineT0, T1, T2), genotype (*LMX1A* with AA or AG/GG; *APOE* with ε4 or Non-ε4), and MCI subtype (aMCI or naMCI) as main effects. Sex, age at baseline, SES, and education were included as covariates. Age and sex were removed from the final model when no significant effects from these variables were found. Possible interactions between the training effect^∗^genotype, training^∗^MCI subtype, MCI subtype^∗^genotype and training^∗^MCI subtype^∗^genotype, were also evaluated. *Post hoc* analyses were performed by the used of paired *t*-tests, comparing either the results at 4 weeks post-training to baseline (T1 vs. T0), 16 weeks post-training to baseline (T2 vs. T0), or T2 vs. T1. For all analyses, since we had *a priori* hypotheses, *p*-values < 0.05 was considered significant, but we additionally calculated the Benjamini-Hochberg false discovery rate (FDR), with a *Q*-value set at 0.05, to assess for those that remained significant.

## Results

**FIGURE 1:**
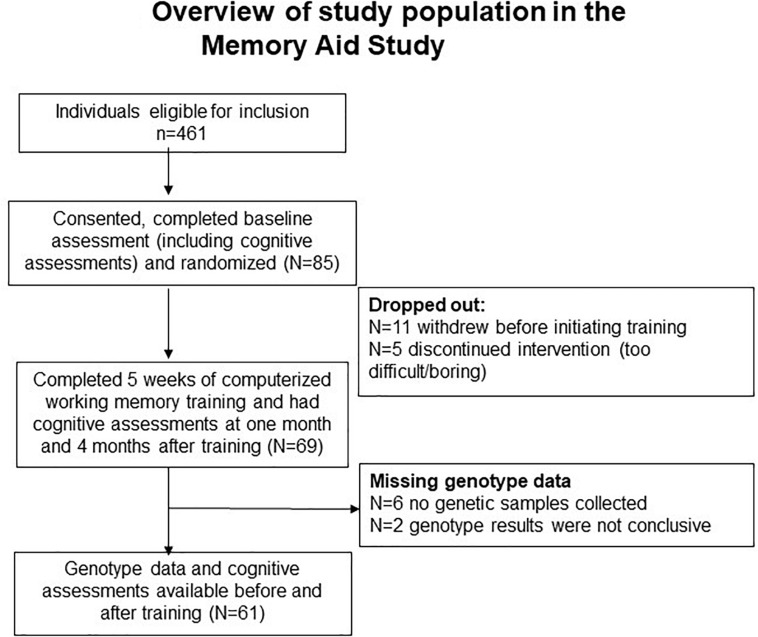
Overview of study population in the memory aid study.

As shown in Figure 1, although 491 participants were eligible for recruitment form these clinics, only 85 participants were recruited from the four centers in the Memory Aid study and provided initial consents to be in the study. Of these 85 participants, 11 individuals changed their minds and withdrew from the study prior to initiating the Cogmed training, leaving 69 (72%) individuals who completed the cognitive training. Blood samples were collected from 63 individuals who provided the additional consents required for the genotyping, but usable genetic data were available only from 61 of these individuals (Figure 1). Only one individual missed the T1 follow-up evaluation, and one individual missing the T2 visit. Based on the screening evaluations and the in-person baseline and follow-up cognitive and neuropsychological evaluations, none of the participants in the current study had moderate or severe depressive symptoms. See Table 2 for the baseline characteristics of the participants.

**TABLE 2:** Participant characteristics, *LMX1A* genotype, *APOE* epsilon (ε) allele, and baseline cognitive performance.

	Total sample (*n* = 61)	Amnestic MCI (*n* = 31)	Non-amnestic MCI (*n* = 30)	*p*/*X*^2^-value
Sex (M/F)	41 M/20 F	23 M/8 F	18 M/12 F	0.24
Age, years (SD)	66 (9)	68 (9)	65 (9)	0.21
Race (White/African/other)	(60/1/0)	(31/0/0)	(29/1/0)	0.29
Education, years (SD)	13 (3)	13 (3)	13 (3)	0.47
SES median (range)	3.3 (4)	3.2 (4)	3.3 (4)	0.64
LMX1A genotype (AA or AG/GG)	37 AA (60.6%) 24 AG/GG	21 AA (67.7%) 10 AG/GG	16 AA (53.3%) 14 AG/GG	0.16
*APOE* ε (ε4 or non-ε4)	27 ε4 (44.3%) 34 non-ε4	15 ε4 (48.3%) 16 non-ε4	12 ε4 (40%) 18 non-ε4	0.36
*APOE* ε (ε4 or non-ε4)/LMX1A genotype (AA or AG/GG)	15 ε4/AA 12 ε4/AG-GG 22 non-ε4/AA 12 non-ε4-/AG-GG	8 ε4/AA 7 ε4/AG-GG 13 non-ε4/AA 3 non-ε4/AG-GG	7 ε4/AA 5 ε4/AG-GG 9 non-ε4/AA 9 non-ε4/AG-GG	0.16
Single domain	32 (52.5%)	8 (25.8%)	24 (80%)	
Multi domain	29 (47.5%)	23 (74.2%)	6 (20%)	
Training type (adaptive/fixed low level)	32/29	15/16	17/13	0.52
**Baseline *z*-scores**				
Working memory domain	0.03(0.84)	−0.20(0.75)	0.27(0.87)	0.05
Attention domain	0.01(0.58)	−0.15(0.46)	0.17(0.64)	**0.04**
Processing speed domain	0.033(0.94)	−0.12(1.2)	0.19(0.65)	0.21
Visual learning short delay domain	0.037(0.68)	−0.31(0.62)	0.38(0.56)	0.00*
Visual memory long delay domain	−0.02(0.84)	−0.46(0.72)	0.43(0.70)	0.00*
Verbal learning short delay domain	0.07(0.88)	−0.54(0.65)	0.65(0.66)	0.00*
Verbal memory long delay domain	0.06(0.89)	−0.61(0.62)	0.69(0.60)	0.00*
Verbal memory recognition domain	0.06(0.96)	−0.19(1.2)	0.32(0.50)	0.08
Executive function domain	0.02(0.79)	−0.16(0.82)	0.22(0.73)	0.**03**

*^∗^Significant after FDR corrections.*

**p*-Values are from unpaired *t*-tests or chi-square between the amnestic MCI and non-amnestic MCI groups.*

*MCI, mild cognitive impairment.*

### MCI Type Effects

At baseline, the aMCI and the naMCI groups had similar age, sex, years of education, race-distribution, SES, and the proportion with the *LMX1A*-AA genotype or the *APOE*ε4 allele (type effects, Table 2). The training type allocation were equally divided between MCI subtype (χ^2^ = 0.52). The two MCI subgroups also showed similar proportion of participants with the combined genotypes of *LMX1A*-AA and *APOE*ε4 allele. Across all MCI participants, 52.5% had single domain cognitive deficits; however, majority (74.2%) of the aMCI group had multidomain deficits while 80% of the naMCI group had single domain deficits. Therefore, as expected, the two MCI-subtype groups differed significantly in all cognitive domains, except for WM, Processing Speed, and Verbal Memory recognition. While the aMCI group performed below the mean, the naMCI group performed slightly above the mean in all domains. Hence, except for Processing Speed, the naMCI performed better than the aMCI group in all domains at all time points (Tables 2, 3, MCI Type Effect). Regarding the WM training type performed, the proportion for each training type were not different between the two MCI groups (aMCI group: 16 had the non-adaptive (fixed level) and 15 had the adaptive training; naMCI group: 13 received the non-adaptive (fixed low level) and 17 had the adaptive training). The two training types were combined since they showed no difference in the training effects.

**TABLE 3:** Visit effects, MCI type effects, and visit*MCI type effects across all subjects.

	Amnestic MCI		Non-amnestic MCI		MCI type effect (*p*-value)	Training effect (*p*-value)	MCI type *training effect (*p*-value)	Amnestic MCI		Non-amnestic MCI	
					
Domain	4 weeks	16 weeks	4 weeks	16 weeks				4 weeks (T1 vs.T0)	4 weeks (T16 vs.T0)	4 weeks (T1 vs.T0)	16 weeks (T2 vs.T0)
	(T1–T0)	(T2–T0)	(T1–T0)	(T2–T0)							
	Mean	Mean	Mean	Mean				Paired-*t* (*p*-values)	Paired-*t* (*p*-values)	Paired-*t* (*p*-values)	Paired-*t* (*p*-values)
	change	change	change	change							
	(SD)	(SD)	(SD)	(SD)							
Working memory	0.33 (0.55)	0.16 (0.70)	0.43 (0.49)	0.25 (0.75)	0.003*	0.000*	0.481	0.004*	0.404	0.000*	0.055
Attention	0.00 (0.41)	−0.01(0.60)	0.12 (0.49)	0.26 (0.40)	0.000*	0.236	0.002*	0.802	0.382	0.129	0.000*
Processing speed	−0.01(0.18)	−0.11(0.24)	−0.08(0.38)	−0.08(0.45)	0.180	**0.034**	0.574	0.807	0.005*	0.272	0.411
Visual learning short delay	−0.05(0.60)	−0.01(0.57)	0.14 (0.34)	0.32 (0.52)	0.000*	0.130	0.123	0.776	0.906	0.098	0.003*
Visual memory long delay	0.08 (0.42)	0.07 (0.81)	0.28 (0.90)	0.42 (0.92)	0.000*	0.053	0.213	0.752	0.391	0.009*	**0.035**
Verbal learning short delay	0.06 (0.50)	0.02 (0.50)	0.05 (0.51)	0.12 (0.56)	0.000*	0.641	0.434	0.954	0.699	0.278	0.182
Verbal memory long delay	0.13 (0.48)	0.15 (0.49)	0.12 (0.51)	0.16 (0.57)	0.000*	**0.049**	0.823	0.226	0.201	0.115	0.095
Verbal memory recognition	−0.31(1.09)	−0.19(1.18)	0.07 (0.67)	−0.05(0.60)	0.001*	0.465	0.163	0.101	0.199	0.472	0.806
Executive function	−0.14(0.30)	−0.19(0.31)	0.07 (0.36)	0.06 (0.36)	0.003*	0.344	0.000*	0.004*	0.002*	0.073	0.127

Bolded *p*-values are ≤0.05; * indicate *p*-values that remained significant after FDR correction.

### Training Effects

Individual training effects in this study were defined as changes in cognitive scores in each domain as compared to baseline. Significant training-related improvements were found in WM, and trends for training effects on Processing Speed and Verbal_Memory_Long-Delay domains across all subjects (Training Effect, Table 3). *Post hoc* analyses and Table 3 showed that, compared to T0, significant improvement was observed in the WM domain at T1 for both the aMCI group (+0.33; *p* = 0.004) and for the naMCI group (+0.43, *p* < 0.00001), but only the naMCI group maintained the improvement (T2 vs. T0: +0.24%, *p* = 0.05). However, in the Processing Speed domain, both groups showed slight declined in performance after WMT, and the aMCI group showed significant decline at T2 (−0.11; *p* = 0.005).

Furthermore, the naMCI group showed greater training effect than the aMCI group (training^∗^MCI type, Table 3) in the Attention (*p* = 0.002) and Executive Function (*p* = 0.0003) domains. *Post hoc* analyses showed these group differences were due to the significant improvement in the Attention domain only in the naMCI group at T2 (+0.26; *p* = 0.003), but significant decline in the Executive function domain only the aMCI group at both T1 (−0.14; *p* = 0.004) and T2 (−0.19; *p* = 0.002).

### LMX1A Genotype Effect

**FIGURE 2:**
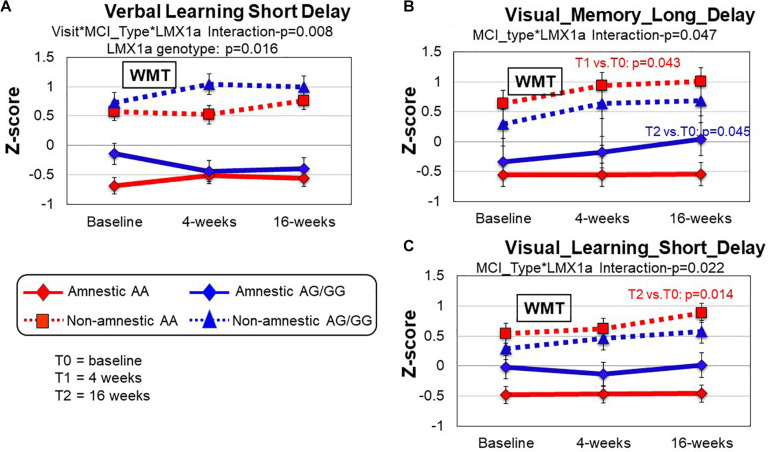
Group comparisons (MCI type and *LMX1A* genotype) of cognitive performance between baseline, 4 and 16 weeks after working memory training (WMT). **(A)** In the Verbal_Learning_Short_Delay domain, the non-amnestic MCI (naMCI) group performed better than the amnestic MCI (aMCI) group (MCI type, *p* = 0.000). The *LMX1A*-AA carriers, regardless of MCI type, consistently had lower scores across all timepoints (Visit*MCI type* *LMX1A*, *p* = 0.008). **(B)** In the Visual_Memory_Long_Delay domain, the naMCI group also tended to perform better than the aMCI group (MCI type, *p* = 0.000). The naMCI group with *LMX1*A-AA also tended to have higher z-scores than the aMCI group across all time points (MCI type*LMX1A, *p* = 0.047). Pairwise comparisons within each group revealed significant improvement at T1 compared to T0 (*p* = 0.043) in the naMCI-*LMX1*A-AA group and at T2 compared to T0 in the aMCI-*LMX1*A-AG/GG group. **(C)** In the Visual_Learning_Short_Delay domain, the naMCI group performed better than the aMCI group (MCI type, *p* = 0.000), and the naMCI group with *LMX1A*-AA had higher *z*-scores than the aMCI group across all time points (MCI type*LMX1A, *p* = 0.022), with significant improvement at T2 compared to T0 (*p* = 0.014). *P*-values are from the inverse proportional weighting, using the generalized estimating equations (GEE) method (see “Statistical Analysis” section for details).

A *LMX1A* genotype effect was found in the Verbal_Learning_Short-Delay domain (*p* = 0.016); within each MCI subtype, those with *LMX1A*-AA genotype had lower performance across all timepoints (Figure 2A). However, in this same domain, a 3-way interaction (training^∗^MCI type^∗^LMX1A, Table 4) showed that those with *LMX1A*-AG/G had improved performance at T1 if they were naMCI, but declined function if they were aMCI subtype. Furthermore, two-way interactions (MCI type^∗^*LMX1A*) were found in the Visual_Memory_Long_Delay and the Visual Learning_Short_Delayed domains (Figures 2B,C). In both of these domains, the naMCI group with *LMX1A*-AA had higher z-scores than the aMCI group across all time points.

**TABLE 4:** Visit effects, MCI type effects, *LMX1A* genotype (AA vs. AG/GG) effects and interaction effects across groups.

Cognitive domain	Amnestic MCI (changes in *Z*-scores)				Non-amnestic MCI (changes in *Z*-scores)				WGEE model (*P*-values)					
			
	LMX1A-AA (*n* = 21)		*LMX1A*-AG/GG (*n* = 10)		*LMX1A*-AA (*n* = 16)		*LMX1A* AG/GG (*n* = 14)		Main effects			2-way interactions		3-way interactions
						
	After	After	After	After	After	After	After	After	Training effect	MCI type effect	LMX1A genotype effect	Training*LMX1A genotype	MCI type* LMX1A genotype	Training*MCI type*LMX1A genotype
	4 weeks	16 weeks	4 weeks	16 weeks	4 weeks	16 weeks	4 weeks	16 weeks						
	(T1–T0)	(T2–T0)	(T1–T0)	(T2–T0)	(T1–T0)	(T2–T0)	(T1–T0)	(T2–T0)						
Working memory	0.46 (0.55)	0.29 (0.71)	−0.00(0.44)	−0.18(0.57)	0.33 (0.53)	0.07 (0.59)	0.55 (0.43)	0.44 (0.88)	0.000*	0.003*	0.431	0.950	0.828	0.341
Attention	0.05 (0.44)	0.07 (0.66)	−0.11(0.33)	−0.21(0.49)	0.02 (0.49)	0.16 (0.45)	0.25 (0.47)	0.38 (0.31)	0.226	0.000*	0.272	0.671	0.643	0.133
Processing speed	−0.02(0.18)	−0.13(0.21)	0.01 (0.21)	−0.07(0.31)	−0.08(0.46)	−0.07(0.44)	−0.07(0.25)	−0.10(0.48)	**0.039**	0.177	0.238	0.981	0.317	0.851
Visual learning short delay	−0.06(0.64)	−0.04(0.61)	−0.05(0.53)	0.05 (0.49)	0.14 (0.37)	0.41 (0.53)	0.14 (0.32)	0.21 (0.51)	0.101	0.000*	0.453	0.937	**0.022**	0.486
Visual memory long delay	0.11 (0.46)	0.01 (0.91)	0.03 (0.34)	0.25 (0.40)	0.15 (1.02)	0.42 (0.73)	0.44 (0.74)	0.41 (1.13)	0.050	0.000*	0.683	0.652	**0.047**	0.775
Verbal learning short delay	0.23 (0.43)	15 (0.31)	−0.35(0.45)	−0.36(0.71)	−0.13(0.46)	0.08 (0.56)	0.28 (0.49)	0.17 (0.58)	0.653	0.000*	**0.016**	0.344	0.948	0.008*
Verbal memory Long delay	0.18 (0.48)	0.19 (0.43)	0.00 (0.49)	0.05 (0.63)	−0.06(0.49)	0.11 (0.49)	0.33 (0.48)	0.23 (0.66)	0.055	0.000*	0.100	0.495	0.538	0.445
Verbal memory recognition	−0.10(0.91)	−0.13(1.34)	−0.79(1.36)	−0.33(0.62)	−0.06(0.82)	0.03 (0.69)	0.24 (0.40)	−0.14(0.49)	0.464	0.001*	0.233	0.725	0.465	0.239
Executive function	−0.18(0.31)	−0.19(0.26)	−0.03(0.25)	−0.19(0.45)	−0.03(0.21)	0.02 (0.25)	0.18 (0.47)	0.10 (0.47)	0.418	0.003*	0.131	0.088	0.975	0.498

Bolded *p*-values are ≤0.05; * indicate *p*-values that remained significant after FDR correction.

### APOE-ε Allele Effect

**FIGURE 3:**
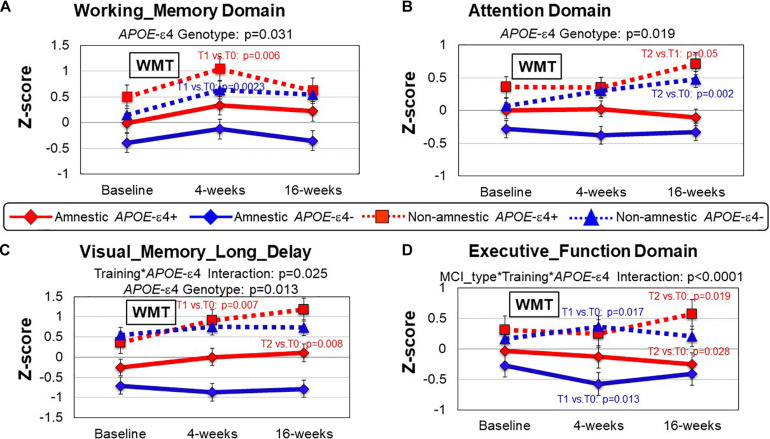
Group comparisons (MCI type and *APOE*ε genotype) of cognitive performance between baseline, 4 and 16 weeks after working memory training (WMT). **(A)** In the Working Memory domain the *APOE*-ε4 carriers (red lines) performed better than the non-carriers (blue lines); *APOE*ε4 genotype, *p* = 0.031. *Post hoc* analyses showed that both the naMCI groups improved at T1 compared to T0 after WMT (*APOE-*ε4: *p* = 0.006; non-*APOE-*ε4: *p* = 0.0023). **(B)** In the Attention domain the naMCI groups (dotted lines) performed better than the aMCI groups (solid lines) at all timepoints regardless of *APOE*-ε4 carriage (MCI type, *p* = 0.019). Furthermore, the naMCI subjects improved further at T2 (compared to T1 for *APOE-*ε4: *p* = 0.05; compared to T0 for *APOE-*ε: *p* = 0.002). **(C)** In the Visual Memory Long Delay domain the *APOE* ε4 groups (red) performed better than the groups without the *APOE* ε4 (blue) across all time points (*APOE* ε4 Genotype, *p* = 0.013), and the *APOE* ε4-carrier groups also showed greater training effects than the groups without the *APOE* ε4 (*APOE* ε4 genotype*training: *p* = 0.025; red lines). *Post hoc* analyses showed that both *APOE-ε4* carrier groups improved further at T2 (T2 vs. T0: naMCI: *p* = 0.007; aMCI *p* = 0.008). **(D)** In the Executive Function domain, the naMCI group performed better than the aMCI group across all time points, and the *APOE* ε4 group showed greater training effect only if they were also naMCI subjects (MCI type*training*APOEε4 genotype, *p* < 0.0001). *Post hoc* analyses demonstrate this at 16 weeks after Cogmed training (T2), while the naMCI patients with *APOE*-ε4 showed improvement in Executive Function (T2 vs. T0, *p* = 0.019), aMCI patients with *APOE*-ε4 showed declined in this domain (T2 vs. T0, *p* = 0.028). The aMCI-*APOE*-ε4 showed declined even at 4 weeks after WMT (*p* = 0.013). *P*-values are from the inverse proportional weighting, using the generalized estimating equations (GEE) method (see “Statistical Analysis” section for details).

Consistent with findings above, regardless of *APOE*-ε4 allele, naMCI patients consistently performed better on all domains across the time points than aMCI patients (MCI Type Effect, Table 5). However, regardless of MCI type, patients with *APOE*-ε4 allele tended to performed better on four cognitive domains than those without *APOE*-ε4: WM (*p* = 0.031), Attention (*p* = 0.019), Processing Speed (*p* = 0.049) and Visual Memory long_delay (*p* = 0.013) (Table 5 and Figures 3A–C, data not shown for Processing Speed). Furthermore, a training^∗^*APOE*-ε4 genotype interaction was found for Visual_Memory_Long_Delay (*p* = 0.025); *APOE*-ε4 individuals showed improved performance after training, but not those without the *APOE*-ε4, regardless of MCI subtype (Figure 3C). Lastly, a 3-way interaction between WMT^∗^MCI type^∗^*APOE*-ε4 genotype was observed for the Executive Function domain (*p* < 0.0001, Table 5). Specifically, at 16 weeks after Cogmed training (T2), while the naMCI patients with *APOE*-ε4 showed improvement in Executive Function (T2 vs. T0, *p* = 0.019), aMCI patients with *APOE*-ε4+ showed declined in this domain (T2 vs. T0, *p* = 0.028), Figure 3D. Th-is 3-way interaction for Executive Functions remained significant after FDR correction (Table 5).

**TABLE 5:** Visit effects, MCI type effects, *APOE*-epsilon allele (ε4 vs. non-ε4) effects and interaction effects across groups.

Cognitive domain	Amnestic MCI (changes in *Z*-scores)				Non-amnestic MCI (changes in *Z*-scores)				WGEE model (*P*-values)					
			
	*APOE* ε4 (*n* = 15)		*Non-APOE* ε4 (*n* = 16)		*APOE* ε4 (*n* = 12)		*Non-APOE* ε4 (*n* = 18)		Main effects			2-way interactions		3-way interactions
						
	After	After	After	After	After	After	After	After	Training effect	MCI type effect	APOEε allele effect	Training*APOEε allele	MCI type*APOEε allele	Training*MCI type*APOEε allele
	4 weeks	16 weeks	4 weeks	16 weeks	4 weeks	16 weeks	4 weeks	16 weeks						
	(T1–T0)	(T2–T0)	(T1–T0)	(T2–T0)	(T1–T0)	(T2–T0)	(T1–T0)	(T2–T0)						
Working memory	0.35 (0.59)	0.21 (0.83)	0.31 (0.54)	0.12 (0.57)	0.51 (0.47)	0.10 (0.89)	0.38 (0.51)	0.33 (0.67)	0.000*	0.003*	**0.031**	0.445	0.553	0.233
Attention	−0.01(0.37)	−0.14(0.63)	0.01 (0.46)	0.11 (0.54)	0.04 (0.62)	0.29 (0.40)	0.17 (0.39)	0.25 (0.41)	0.224	0.000*	**0.019**	0.806	0.604	0.247
Processing speed	−0.02(0.23)	−0.09(0.31)	−0.00(0.13)	−0.14(0.15)	−0.02(0.22)	−0.01(0.29)	−0.11(0.45)	−0.09(0.53)	**0.039**	0.179	**0.049**	0.827	0.945	0.438
Visual learning short delay	0.08 (0.48)	0.07 (0.49)	−0.18(0.69)	−0.09(0.64)	0.16 (0.31)	0.40 (0.49)	0.12 (0.37)	0.27 (0.55)	0.098	0.000*	0.053	0.413	0.096	0.748
Visual memory long delay	0.21 (0.44)	0.31 (0.44)	−0.04(0.38)	−0.14(0.99)	0.58 (0.49)	0.73 (0.83)	0.09 (1.05)	0.24 (0.94)	**0.047**	0.000*	**0.013**	**0.025**	0.092	0.887
Verbal learning short delay	0.09 (0.52)	0.05 (0.59)	−0.04(0.50)	−0.02(43)	0.05 (0.47)	0.11 (0.73)	0.05 (0.54)	0.13 (0.46)	0.664	0.000*	0.085	0.923	0.163	0.757
Verbal memory long delay	0.06 (0.57)	0.10 (0.61)	0.20 (0.38)	0.20 (0.36)	−0.08(0.59)	0.16 (0.65)	0.23 (0.46)	0.17 (0.51)	0.064	0.000*	0.336	0.073	0.552	0.432
Verbal memory recognition	−0.64(1.37)	−0.60(1.08)	0.02 (0.57)	0.20 (1.17)	−0.11(0.97)	0.13 (0.39)	0.19 (0.41)	−0.16(0.68)	0.458	0.001*	0.950	0.124	0.201	0.295
Executive function	−0.10(0.28)	−0.24(0.33)	−0.17(0.39)	−0.15(0.30)	−0.06(0.19)	0.16 (0.32)	0.14 (0.42)	−0.00(0.38)	0.405	0.003*	0.176	0.508	0.621	0.000*

Bolded *p*-values are ≤0.05; * indicate *p*-values that remained significant after FDR correction.

## Discussion

The main findings of the present study are: (1) The naMCI group performed better in majority of the cognitive domains compared to the aMCI group at baseline. (2) All participants showed improved cognitive performance on several domains (Working Memory, Processing Speed, and Verbal_Memory_Long_Delay) after WM training. The naMCI group showed greater training effects than the aMCI group on Attention and Executive function. After 16 weeks, the naMCI group was able to maintain their training gains in WM and showed further improvement in Attention, while the aMCI group showed significant decline in these domains. (3) For the *LMX1A* genotype, naMCI patients with AA genotype had better training effects than those with AG/GG genotypes on Verbal_Learning_Short_Delay and Visual_Learning_Short_Delay. (4) Our participants tended to show better cognitive performance in WM, Attention, Processing Speed, and Visual_Memory_Long_Delay only if they were *APOE*-ε4 carriers. Lastly, only those with naMCI and the *APOE*-ε4 carriers showed improved Executive Function after 16 weeks of training. Collectively, these findings demonstrate how the subtype of MCI, and their *LMX1A* genotype or presence of *APOE*-ε4 allele, may influence cognitive training outcomes, which would be important in designing the optimal training program for individuals with these different genotypes.

The poorer cognitive performance in the aMCI group compared to the naMCI group is consistent with previous research (Petersen et al., 1999, 2017; Jack et al., 2016; Ten Kate et al., 2017). Patients with aMCI also showed much greater prevalence of positive amyloid PET imaging (with carbon-11-Pittsburgh compound B), and were more likely to progress to Alzheimer’s disease dementia compared to naMCI patients (Oltra-Cucarella et al., 2018; Jimenez-Bonilla et al., 2019). These findings suggest that the level of neural plasticity or cognitive reserve might be reduced with disease progression and increasing amyloid deposition in the aMCI patients but relatively preserved or less affected in naMCI patients. Therefore, although all participants in this study showed improved WM and Verbal_Memory_Long_Delay after the Cogmed training at the 1-month follow-up, only the naMCI group maintained their training gain in WM and further improved on Attention at 16 weeks, while the aMCI subjects showed continued decline in Attention and in Processing Speed at follow-ups. The improved WM after Cogmed training is similar to previous studies (Belleville et al., 2006; Rozzini et al., 2007; Talassi et al., 2007), while the improved Verbal_Memory_Long_Delay represents a transfer of training effect to a non-trained domain. The greater decline in Processing Speed in the aMCI group than the naMCI group might also be viewed as a disease marker, possibly linked to reduced connectivity based on impaired neural and white matter integrity (Park and Reuter-Lorenz, 2009).

At 16 weeks after training, the naMCI groups showed significant improvements in Attention and Executive Function, while the aMCI group showed progressive decline in Executive Function. Attentional abilities correlated with independent living in aMCI, but not in naMCI (Putcha and Tremont, 2016). Our findings in the naMCI patients are consistent with those in MCI patients with small-vessel disease who showed improved attention and executive function after a targeted training in attention (Pantoni et al., 2017). Furthermore, the higher baseline cognitive function in the naMCI group likely contributed to the greater training effects since they might have more cognitive reserve and neuroplasticity. Although our aMCI group did not show improvement in Attention, they remained stable for the duration of the study, which may reflect a type of training effect to remain stable, given their likely reduced cognitive reserve and neural plasticity. Another study of MCI patients that focused on “executive attention” training found improvement only in selective attention (Digit Span Task, same task as in our WM domain) (Yang et al., 2019), without transfer effects to “focused attention” (Stroop Color Word Test, same as in our Executive Function domain). However, their study population were older which might have impacted the results. They also did not separate the MCI patients into aMCI and naMCI which might have confounded their findings.

Another variable that might influence the training effect or group differences in the baseline cognitive performance is the polymorphism of the dopaminergic gene *LMX1A*, since those with the AA genotype showed greater training effects than those with the AG/GG genotypes after WM training, both in healthy individuals (Bellander et al., 2011) and in those with HIV-infection (Chang et al., 2017). In contrast to these earlier studies, we did not find greater training effects in WM; instead, only naMCI patients with *LMX1A*-AA showed greater training gains in Verbal Learning Short_Delay, and trends for greater training effects in Verbal Memory and Visual Learning domains as well. The *LMX1A* gene encoded protein is a transcription factor that regulates insulin gene transcription, and maintains mitochondrial function in midbrain dopaminergic neurons (Doucet-Beaupre et al., 2016). Hence, this essential protein maintains the survival of dopaminergic neurons, which are involved not only in motor function, but also in motivation, learning and memory. Dopaminergic receptors mediate WM (Goldman-Rakic, 1996b) and those with *LMX1A-*AA genotype showed greater improvement and neural efficiency after Cogmed training (Chang et al., 2017). However, recent data demonstrated that dopaminergic function may also impact hippocampal memory processes (Chowdhury et al., 2012) with less specific memory retrieval in older adults due to the dedifferentiation of cognitive aging (Abdulrahman et al., 2017). Since verbal learning is thought to be a sensitive marker for progression from memory impairment to dementia (Bondi and Smith, 2014), the trainability of this domain might be an important target for further research. In our study, we were only able to find a training gain for verbal learning in the naMCI LMX1A-AA group 1-month after WM training. Lastly, similar to our study, carriers of the val allele of the COMT Val^158^Met polymorphism, another gene that regulates the dopaminergic system, also showed lower baseline performance but greater plasticity of working memory (Bellander et al., 2015), and showed greater WM training-related prefrontal plasticity (Zhao et al., 2020). Future studies should include the evaluation of COMT polymorphism.

Another consideration is the prevalence of the *LMX1A*-AA genotype in each of the MCI subgroups. The *LMX1A*-AA allele for the rs4657412 SNP in our current study is 60.6% for the total sample, 67.7% for the amnestic MCI group and 53.3% for the non-amnestic MCI group (Table 2), which are similar to this allelic frequency in the general population from two Swedish cohorts that showed the frequencies of 54.2 and 61.8% (Bergman et al., 2010; Rolstad et al., 2015). In Parkinson’s disease, the allelic frequency of the A allele for the rs4657412 SNP was 24%, which was marginally higher than the controls at 20.7% (Bergman et al., 2009). Despite the slightly increased prevalence of the *LMX1A*-AA genotype in the aMCI group (67.7%) than the naMCI group (53.3%), they did not benefit from having this genotype and showed a poorer WMT effect than the naMCI group. Furthermore, polymorphism of *APOE* genotype may influence the training outcomes. *APOE* is a protein required for trophic support, programmed cell death, microtubule disassembly, synaptic function, aging, and insulin resistance—all processes that have been implicated in AD pathogenesis (Theendakara et al., 2018). *APOE*-ε4 carrier status was associated with greater memory impairment in analyses that co-varied for duration of disease (Smith et al., 1998). In studies that combined AD dementia and MCI, ε4 homozygosity was associated with poorer retention, learning, and verbal comprehension at a given disease duration (Smith et al., 1998). The progression from MCI to AD was also found to be faster in homozygotic carriers than in the carriers of one or no *APOE*-ε4 allele (Tuminello and Han, 2011). Currently, no known knowledge exists whether targeted cognitive interventions may benefit those with increased genetic risk of cognitive impairments. However, in the current study, *APOE-*ε4 carriers tended to perform better on WM, Attention, Processing Speed and Visual Memory Long_Delay, and only the *APOE*-ε4 carriers, regardless of MCI type, showed improvements in the Visual Memory-Long Delay domain after the Cogmed training. These findings are consistent with the antagonistic pleiotropy effects of *APOE*-ε4, since our MCI patients are relatively younger than the typical AD patients, and may still be able to utilize their cogntive and neural reseve (Tuminello and Han, 2011; Chang et al., 2016). Compared to the typical Alzheimer’s disease (AD) patients, our MCI patients were relatively younger; differences in age likely contributed to these diverging results on the effects of *APOE*-ε4 on cognitive performance. Since those with *LMX1A*-AA genotype or *APOE*-ε4 allele showed better cognitive outcomes after the WMT, we might expect individuals with both of these genotypes to show the best outcomes. However, given the small sample sizes and similar proportion of this combination in either the aMCI (*n* = 7) or naMCI (*n* = 8) group, we could not determine this possible outcome. A future larger study is needed to evaluate whether this combination would lead to the strongest training effects, or would modulate the training effects differently in aMCI versus naMCI patients.

Furthermore, over the follow-up period of almost 6 months (from baseline to 16 weeks after the Cogmed training), the opposing effect from disease progression might hide additional training gains especially in the aMCI carriers. The *APOE-*ε4 carriers may be able to compensate more, both in magnitude and extent in neuronal activation, than the non-carriers by recruitment of their inferior frontal gyrus in the prefrontal cortex during challenging WM tasks (Scheller et al., 2017). In the Executive Function domain, the naMCI *APOE* ε4-carrier group showed a significant improvement after 16 weeks, whereas the aMCI *APOE* ε4-carrier group showed significant decline. This disparate effect might be due to the greater underlying amyloid deposition and possibly lesser cognitive reserve in the aMCI patients, compared to those with naMCI.

### Limitations

Despite the encouraging findings in the current study, several limitations should be considered to improve future studies in patients with MCI. First, the sample size for each of the subgroups is relatively small, especially when the modulating effects of the genotypes on complex traits such as cognitive functions are investigated. Given the small sample in the subgroups, these findings must be interpreted with caution and should be considered preliminary to guide future larger validation studies. Furthermore, although we would expect that the naMCI participants who had the combined genotypes of *LMX1A*-AA and *APOE*-ε4 would perform even better than those without this combination of genotypes after the WM training, the samples size was too small for us to draw any conclusions and will need to be investigated in a future larger study, Second, the study participants are mainly of Scandinavian descent, which minimized the genetic heterogeneity, but the findings may not be generalizable to other racial groups. For instance, racial disparity exists for dementia and the risk gene *APOE*-ε4 doubles the risk for dementia among whites with no increase among blacks (Weuve et al., 2018). Third, the participants in the current study had twice as many men than women, which is not typical for individuals with MCI or AD. One reason for the greater proportion of men than women in our study might be the fact that more men used computers than women in these regions of Norway, and thus were more likely to participate in this computer-training study. Therefore, our findings may not be generalizable to the typical MCI population. Future larger studies need to enroll a representative sex-proportion of individuals with MCI. Fourth, a selection bias in “help-seeking” attitudes might be present within the study population, as the participants were recruited from the memory clinics and were mainly well educated and motivated to improve their cognition.

However, a major strength of our study is the use of a well-defined MCI definition from a Memory clinic sample, which also minimized the variability in the study sample. Furthermore, all patients were assessed by the same experienced neuropsychologist, which eliminated the inter-tester variability. The efficacy of computerized cognitive training in MCI patients is debated (Sherman et al., 2017), mostly due to variable results, poor scientific quality on some studies and commercial marketing that promise benefits to multiple cognitive domains. Our study finds transfer effects in some non-trained domains after WM training but not in all. No consensus identifies the cognitive domains where restorative interventions will have the largest impact on daily life function in MCI patients. This knowledge gap needs to be identified in order to individualize a targeted intervention. WM training in individuals with MCI might be an effective intervention to delay the onset of dementia by increasing the compensatory, neuroplasticity abilities (scaffolds), by targeting WM specifically and cognitive reserve generally. Longitudinal studies that follow MCI patients after cognitive (WM) training is needed to evaluate this possibility.

### Clinical Implications

Currently, no curative or memory restoring interventions exists for individuals with MCI. This study shows preliminary evidence for different effects of WMT that are dependent on MCI subtype, and on the genetic polymorphisms of two of genes. WMT in the naMCI patients showed promising results.

The effects of the *LMX1A* and *APOE* genes follow complicated patterns across the life span, depending on interactions with other genes and background factors. Clinically, a stable and maintained performance in cognitive function over time might reflect an actual training gain since some of the participants might have ongoing neuropathological progression that counteract the effects of training. Future larger and longitudinal studies are needed to validate our findings, and to determine the long-term outcomes.

## Conclusion

Working memory training may improve cognitive function, both for WM and other non-trained domains. Our data indicate that the MCI subtypes and the genotypes may both influence training effects. Carriers of the *APOE*-ε4 allele showed positive cognitive effects from the intervention regardless of the subtype, which suggest that these relatively younger MCI patients still have adequate cognitive reserve or neural plasticity. Therefore, cognitive training programs should consider the MCI subtype, as well as the individual’s genetic information, to facilitate a more personalized training approach.

## Data Availability Statement

The datasets presented in this article are not readily available because The Norwegian Regional Committee for medical and health research ethics limit data sharing, and de-identified data can only be shared after an application process. The study protocol is publicly available. The statistical analysis plan is available upon request by members of the academic community for the next five years. The generated datasets are available by request to the corresponding authors. Requests to access the datasets should be directed to SH, Susanne.sorensen.hernes@sshf.no.

## Ethics Statement

The studies involving human participants were reviewed and approved by The Norwegian Regional Committee for medical and health research ethics, South-Eastern region (2013/410) approved the study (clinicaltrials.gov NCT01991405). The patients/participants provided their written informed consent to participate in this study.

## Author Contributions

SH, MF, GL, JS, and LC conceptualized and designed the study. MF, GL, AE, AP, IU, B-OM, A-BK, TL, and HH collected the data. SH, MF, XZ, and LC analyzed the data. SH, MF, HH, GL, JS, and LC interpreted the data. SH, MF, GL, XZ, and LC drafted the article. All authors have critically revised the article and approved the final version of manuscript to be published.

## Conflict of Interest

The authors declare that the research was conducted in the absence of any commercial or financial relationships that could be construed as a potential conflict of interest.
